# Purdue’s development of abuse-deterrent formulation opioids as a response to market competition: An industry documents analysis

**DOI:** 10.1016/j.ssmqr.2026.100719

**Published:** 2026-03-02

**Authors:** Anthony DiMario, Kelly Knight, Laura A. Schmidt, Dorie E. Apollonio

**Affiliations:** a UCSF Cardiovascular Research Institute, Center for Tobacco Control Research and Education, University of California, San Francisco, CA, USA; b School of Medicine, University of California, San Francisco, San Francisco, CA, USA; c School of Pharmacy, University of California, San Francisco, San Francisco, CA, USA

**Keywords:** Opioid, Analgesic, Regulation, Pharmaceutical industry

## Abstract

**Background:**

The opioid overdose crisis constituted one of the greatest public emergencies in US history. The overpromotion and overprescription of oxycodone and Purdue Pharma’s branded formulation, OxyContin, have been implicated as key drivers of the opioid overdose crisis. This study sought to understand Purdue’s motivations in developing opioid abuse-deterrent formulations, drugs designed to reduce misuse, abuse, and diversion of prescription opioids.

**Methods:**

We conducted a qualitative archival analysis of internal corporate documents archived at the UCSF-JHU Opioid Industry Documents Archive (OIDA). We gathered, coded, and analyzed over 80 internal documents spanning from 1996 to 2010 to reconstruct the timeline of events and motivations that drove Purdue’s development and eventual release of an abuse deterrent formulation OxyContin in the US. These primary data included emails, slideshows, and other textual and visual documents. We used regulatory documents and court records as a supplementary data source to triangulate and validate our OIDA-based findings.

**Results:**

Purdue initially proposed the development of an abuse-deterrent formulation of OxyContin as a strategy to protect its patents and prevent the introduction of a competing generic formulation of oxycodone. When the company successfully litigated against the introduction of a generic medication, it stopped attempting to develop a reformulation until the regulatory environment changed in a way that would prevent competition if Purdue released the reformulated OxyContin.

**Conclusions:**

Pharmaceutical companies may focus on strategies such as abuse-deterrent formulations to protect market share rather than to protect public health. Regulators should be aware of the potential risks of encouraging this approach.

## 1. Introduction

The opioid overdose crisis constituted one of the greatest public emergencies in US history. The semi-synthetic opioid Oxycodone and Purdue Pharma’s promotion of its branded formulation OxyContin have been implicated, through the overpromotion and overprescription of these drugs, as key drivers of the opioid overdose crisis (Hedgpeth, 2023; Kibaly et al., 2021; Marie & Noble, 2023). From 1999 to 2023, over 800,000 people in the US died from an opioid overdose. In 2015, for the first time in a century, life expectancy in the US declined, a decrease largely attributed to an increase in opioid-related mortality (DeWeerdt, 2019). The crisis is complex, involves both prescription and illicit opioids, has multiple drivers, and continues to create disparities by race, class, and geography in terms of morbidity and mortality (Beletsky & Davis, 2017; Ciccarone, 2019, 2021; Dasgupta et al., 2018; Friedman & Hansen, 2024). Although opioid-overdose mortality in the 2020s was primarily associated with synthetic fentanyl and stimulant co-use, the initial deaths involved prescription opioid misuse (Ciccarone, 2021; Netherland & Hansen, 2017; Quinones, 2015). The US continues to consume opioids at a higher per capita rate than other countries (Cornell et al., 2021).

Scholars, journalists, policymakers, and several class-action lawsuits have documented the role of the pharmaceutical industry in catalyzing and profiting from opioid overuse. The industry accomplished this through multiple means, including misrepresenting the risk of opioids to regulators (Caleb Alexander et al., 2022; Sherman, 2023; van Schalkwyk et al., 2024; Yakubi et al., 2022a), manipulating science and shaping medical education to encourage prescribing to a broader population of consumers (Gac et al., 2023, 2024; Gupta et al., 2024; Sismondo, 2018; Sismondo & Bernisson, 2024), aggressively marketing drugs (Eisenkraft Klein et al., 2024; Haddock et al., 2025; Lee et al., 2024; MacKenzie et al., 2023; Yakubi et al., 2022a, Yakubi et al., 2022b), and denying or deflecting responsibility for misuse and diversion (Kabella et al., 2025; Knight et al., 2017). These efforts were consistent with broad industry strategies intended to mitigate regulatory action and increase the profitability of health-harming products, referred to collectively as commercial determinants of health (Freudenberg et al., 2021; Friel et al., 2023; Gilmore & Peeters, 2013; Kickbusch et al., 2016; Lacy-Nichols et al., 2022; Lee & Freudenberg, 2022; van Schalkwyk et al., 2024). Much of the literature on commercial determinants of health has focused on how industry seeks to influence the public and governments. Less attention has been paid to competitive dynamics between corporations, whether to differentiate products, defend patents, or capture market share, and how they influence both policy and population health.

Although the opioid industry was successful in delaying regulations that would undermine the reach and profitability of their products, it was not immune. Beginning in the early 2000s, federal law enforcement and regulatory bodies became aware of increasing misuse and diversion of prescription opioids (U. S, 2015). During this period, regulators and industry actors negotiated and collaborated on approaches that would allow the industry to address the crisis by restricting access to and increasing the safety of opioids (Kolodny, 2020). Post-market surveillance and regulation constituted one major strategy, including the implementation of risk evaluation and mitigation strategies (REMS) as part of drug labeling (Cepeda et al., 2017; Kabella et al., 2025; Knight et al., 2017; Sherman, 2023). Another strategy was to develop “safer” opioids, specifically, reformulations of existing drugs that included “abuse-deterrent” properties to discourage misuse or diversion. Assessing what constitutes an abuse-deterrent formulation remained challenging into the 2020s (Center for Drug Evaluation and Research, 2021). However, the term is generally applied to a pharmaceutical product designed with the intention to deter misuse or abuse without impacting clinical efficacy (U.S. Food and Drug Administration, 2017). This includes efforts to reduce a drug’s manipulability or likeability for non-clinical users (e.g., preventing crushing, reducing euphoria). For example, with a non-abuse deterrent formulation, a user may be able to crush and snort an extended-release tablet to gain an immediate and intense high, whereas an abuse-deterrent formulation may include properties that prevent this physical manipulation or include an aversive agent to make insufflation unpleasant.

This study sought to understand the context and motivations of opioid manufacturers in developing abuse-deterrent formulations. Specifically, we focused on factors that led to Purdue’s decision to release an abuse-deterrent formulation of OxyContin in 2010, given the distinctive role Purdue played in catalyzing the opioid overdose crisis (Hedgpeth, 2023; Kibaly et al., 2021; Marie & Noble, 2023). Our analysis leverages a unique and strategic data source: formerly confidential internal corporate documents that were released as part of settlement agreements during litigation against opioid manufacturers (Alexander & Tasker, 2024; Caleb Alexander et al., 2022). These data offer a rare window into internal corporate motives, communications, strategy, and activities, which often contrast with those presented to the public and policymakers. Qualitative archival analyses of corporate documents allow researchers to reconstruct timelines of events, evaluate corporatations’ potential motives and strategies, and scrutinize the (mis)alignment of commercial and public interest vis-aa-vis vis-aacompetitive advantage and profit versus public health (Bero, 2003; Caleb Alexander et al., 2022; Gaber et al., 2023; Malone & Balbach, 2000). We utilized established methods of industry documents research, particularly those pioneered through studies of the tobacco industry, to collect our findings and develop our analysis, including using strategic keyword search techniques, snowball sampling, and triangulation using multiple documentary sources (Anderson et al., 2011; Bero, 2003; Malone & Balbach, 2000). Our findings demonstrate that Purdue’s interest in abuse-deterrent formulations was a calculated response to competitive market threats in the form of patent litigation and generic medications promoted by other industry actors, rather than an effort to reduce misuse or diversion. These findings build on the commercial determinants of health literature by engaging how competitive market dynamics might shape product availability and consequently patient health and safety. In doing so, we connect commercial determinants of health with studies of the political economy of the pharmaceutical industry.

## 2. Methods

We conducted a qualitative archival analysis of internal corporate documents archived at the UCSF-JHU Opioid Industry Documents Archive (OIDA). OIDA is a repository of internal documents made public as a result of litigation against opioid manufacturers, distributors, and others involved in bringing potent drugs to consumers (Caleb Alexander et al., 2022). As of February 2026, OIDA held over 6.6 million documents, totaling more than 31 million pages. These include emails, memos, presentations, sales reports, budgets, audit reports, Drug Enforcement Administration briefings, meeting agendas and minutes, expert witness reports, and depositions of company executives (Caleb Alexander et al., 2022; Lee et al., 2024; MacKenzie et al., 2023). OIDA constitutes a unique and strategic data source for social science research on corporate harm. These previously confidential internal documents offer a rare window into internal corporate communications, activities, strategies, and motivations, which often contrast with the messages presented to the public. These data therefore allow scholars, policymakers, and the public to better scrutinize corporate claims of alignment of their organizational interests with that of the public good and public health rather the simply take them at face-value.

Our analysis focused on original documents collected from inside Purdue (e.g., executive correspondence, internal company research), supplemented with public-facing documents and secondary literature. To understand Purdue’s development of an abuse-deterrent formulation, archival documents housed in the OIDA were the primary data source, supplemented by court records and regulatory documents used for triangulation and validation of findings. Secondary literature, including journalistic and academic literature, was used to further support and contextualize findings. We build upon a tradition of industry documents research, which often intersects with but remains distinct from the larger commercial determinants of health literature as well as other archival methods, such as those practiced by historians (Anderson et al., 2011; Apollonio & Malone, 2005, 2010; Caleb Alexander et al., 2022). Industry documents analysis began with studies of the tobacco industry, whose internal documents were similarly released through litigation (Bero, 2003; Glantz, 1996; Malone & Balbach, 2000; Proctor, 2012). Subsequent studies of tobacco, pharmaceutical, food, and chemical industries established best practices for data collection and analysis using these unique data, which include strategic keyword searches, snowball sampling, triangulation, validation, and interpretation (Apollonio, 2022; Apollonio & Malone, 2010; Gaber et al., 2023; Hendlin et al., 2020; Kearns et al., 2018; Steele et al., 2025).

Archival data collection was conducted between November 2024 and April 2025. Initial search terms included “abuse-deterrent,” “abuse-resistant,” and “tamper-resistant,” and included all dates and collections. These preliminary searches returned high volumes of results (~8000 to ~55,000 per search term). OIDA’s search architecture uses an algorithm that organizes results by anticipated relevance to search terms. As a result, the first author initially reviewed the first 20 results for each search term to develop an understanding of terminology, actors, and developments pertinent to abuse-deterrent formulations. This preliminary review suggested that Purdue had considered abuse-deterrent formulation development prior to the FDA’s regulatory intervention in 2010. Given Purdue’s early interest and the company’s outsized role in the opioid crisis, further searches focused on Purdue, as well as McKinsey, a consulting firm hired by the company. Keyword searches on “abuse-deterrent” before 2010 in Purdue-related documents returned 80 documents, dated between 1996 and 2006. These 80 documents were reviewed chronologically and coded for relevant themes, events, and actors. Following established procedures in industry documents research, this subset of documents was then used to drive snowball sampling of other documents and collections (Anderson et al., 2011; Cataldo & Malone, 2008; Smith & Malone, 2009). These included searches for other named actors (e.g., Grünenthal, Endo), technologies and drugs (e.g., OROS, Palladone, OCX), and specific litigation (e.g., the Southern District of New York). When there was uncertainty about the relevance of a document, an additional author reviewed it for validation.

We took a modified abductive approach to data collection and analysis (Tavory & Timmermans, 2014; Timmermans & Tavory, 2012). We iteratively used findings from OIDA searches to inform searches for supplementary and secondary literature, using insights from the commercial determinants of health to drive our interpretation of textual data and using our data to challenge and build upon the commercial determinants of health literature (Alexander & Tasker, 2024; Freudenberg et al., 2021; Friel et al., 2023; Kickbusch et al., 2016). Abductive analysis refers to a qualitative approach that iteratively moves between data and literature to inform hypothesis testing, conceptual development, and theory building (Tavory & Timmermans, 2014; Timmermans & Tavory, 2012). Abductive analysis contrasts with both a purely inductive, grounded approach and more theory-driven approaches to qualitative analysis (for these alternative approaches, see, Burawoy, 1998; Corbin & Strauss, 2014; Levenson & Seim, 2024; Tavory & Timmermans, 2009). Abduction is typically applied to ethnographic and interview data, where the ‘living’ nature of interlocutors as data sources allow for strategic, iterative probing. Archival and documents-based studies allow for no such probing, requiring a modification of this approach to allow for adaptive searches, flexible coding, and integration of supplementary sources to support this iterative process. In this endeavor, we allowed ourselves to be open to unexpected findings, new directions, and novel interpretations and connections to existing literature. Court records, regulatory documents, and secondary literature were used to inform additional searches in OIDA, triangulate findings from internal documents, provide a more comprehensive account of relevant events, and extend the analysis beyond documents contained in OIDA. Documents were summarized and discussed by the authors in regular meetings and categorized into a timeline by the authors working in sequence.

## 3. Results

The results of our archival analysis cover an approximately 30 year period, focusing largely on the years between 1995 and 2010 that is, the years between OxyContin’s original introduction to the US market and its reformulation as an abuse-deterrent, respectively. We begin with the origins of abuse-deterrent formulations (ADF) as a concept and intervention by reviewing the case of Talwin. We then analyze Purdue’s initial release of OxyContin, which paired intensive promotion with aggressive patent litigation. Starting in the year 2000, Purdue became involved in an intensive patent dispute with generic manufacturer Endo. The lawsuit threatened to invalidate several of Purdue’s patents on OxyContin and jeopardize Purdue’s primary revenue stream. During this time, Purdue began a consulting relationship with McKinsey. McKinsey recommended scaling back most research and development activities, except for abuse-deterrent formulations and helped guide Purdue through negotiations with several potential partners, namely the German company Grünenthal, to speed development. Both Purdue and McKinsey viewed abuse-deterrent reformulations as a way to extend patent exclusivity, which in the absence of a regulatory mandate, would not effectively recapture the market share lost to generic competitors. When Purdue won its litigation against Endo in 2006, it slowed development of an ADF until 2009. In 2010, in consultation with the FDA, Purdue pulled the original formulation of OxyContin from the market and replaced it with its abuse-deterrent reformulation, in anticipation of new regulatory guidelines for opioid manufacturers and prescribers.

### 3.1. 1980s: First introduction of an abuse-deterrent formulation in the US

The first effort to develop an abuse-deterrent commercial opioid was Talwin NX, approved by the FDA in 1982. Talwin, a synthetic opioid analgesic used to treat moderate to severe pain and a branded form of pentazocine, was approved by the US Food and Drug Administration (FDA) in 1967 and made available in the United States as a putatively non-addictive opioid analgesic (DeNosaquo, 1969). By the mid-1970s, users discovered that a crushed mixture of Talwin and an HC1 antihistamine could be injected to induce a euphoric high comparable to heroin, a combination often referred to colloquially as *Ts and Blues* (Herzberg, 2012, 2020). In 1979, after growing public and congressional concern, the DEA reclassified pentazocine as a schedule IV-controlled substance following a public petition, effectively removing its over-the-counter availability, while other states individually placed Talwin in the more stringent schedule III category (scheduling is the process used in the IUS to classify drugs by their potential for abuse, ranging from Schedule I—high potential for abuse with no accepted medical use—to Schedule V—lowest potential for abuse and accepted medical uses)(Herzberg, 2020; U.S. Drug Enforcement Administration, n. d.). In 1982, Sterling-Winthrop, the drug’s manufacturer, reformulated Talwin to include 0.5 mg of the opioid antagonist naloxone in order to block pentazocine’s euphoric effects when injected and produce withdrawal-like symptoms in opioid tolerant individuals (Carlson, 1983; Schaeffer, 2012). Given the decline in Talwin-related arrests and emergency room visits, researchers, regulators, and public officials perceived that the reformulation effectively deterred abuse (Baum et al., 1987; Herzberg, 2012, 2020; Lahmeyer & Craig, 1987; Poklis, 1984; Schaeffer, 2012). Misuse of Talwin declined precipitously after the reformulation, though retrospectively it is unclear whether this decline was attributable to the reformulation, the wider availability of cheaper heroin beginning in the 1980s, or a combination of both (Baum et al., 1987; Herzberg, 2020; Lahmeyer & Craig, 1987; Poklis, 1984; Schaeffer, 2012).

### 3.2. 1990s: Purdue begins marketing OxyContin

Purdue Pharma L.P. was founded in 1892 and acquired in 1952 by brothers Arthur, Mortimer, and Raymond Sackler (Keefe, 2021; Purdue Pharma L.P., n. d.). Through the latter half of the 20th century, Purdue steadily re-envisioned itself as a pioneer in pain management through opioid analgesics; its first entrant into this emerging clinical market was MS Contin, a slow-release morphine-based painkiller approved by the FDA in 1987 (Sherman, 2023). The eventual expiration of MS Contin’s patents and new generic competition motivated the development of OxyContin (the company’s branded formulation of oxycodone)(Cohle & Ortega, 2023). Purdue received FDA approval in 1995 and quickly began marketing OxyContin as a revolutionary breakthrough in the pharmaceutical management of pain, claiming that OxyContin’s slower release and less frequent dosing relative to faster-acting opioids would manage chronic and breakthrough pain better with less abuse potential (Van Zee, 2009).

Purdue’s strategy for OxyContin wedded aggressive marketing with aggressive litigation. In an internal email chain dated August 23rd, 1996, Richard Sackler proposed a press release to Michael Friedman, a Purdue marketing and sales executive (and later CEO) that would emphasize OxyContin’s “uses and success in the market”(Sackler, 1996). Sackler stressed how the press release connected with the marketing strategy for OxyContin:

“I want to signal the licensing in the market for the product around the world. [To] get an audience for our patent infringement suits so that we are feared as a tiger with claws, teeth, and balls, and build some excitement with prescribers that OxyContin Tablets is the way to go”

(Sackler, 1996).

Purdue sought to promote OxyContin as critically important to patient care directly to physicians, and to signal to potential competitors that Purdue would litigate to defend its patents. Intensive promotion paired with aggressive patent litigation formed the core of Purdue’s strategy to achieve market dominance and would largely go unchallenged until the early 2000s.

### 3.3. 2000: Endo International proposes a generic alternative to OxyContin

In September 2000, Endo International, a pharmaceutical company focused on producing generic drugs, filed an abbreviated new drug application (ANDA) with the FDA, seeking approval to manufacture and market a generic version of OxyContin (Purdue Pharma LP v. Endo Pharmaceuticals Inc., 2006). In compliance with the Hatch-Waxman Act, legislation governing how generics come to market, Endo certified that it would not market the proposed drug until relevant patents expired, or the relevant patents are “invalid or will not be infringed by the manufacture, use, or sale of the new drug for which the [ANDA] is submitted”(Purdue Pharma LP v. Endo Pharms. Inc, 2004, p. 2). Endo also notified Purdue of its filings. Purdue filed suit against Endo in October 2000, and Endo in turn amended its ANDA twice in 2001 (Purdue Pharma LP v. Endo Pharms. Inc, 2004). The FDA gave Endo tentative approval for select dosage ranges in July 2002, while litigation was pending (Purdue Pharma LP v. Endo Pharmaceuticals Inc., 2006). With the approval of a generic competitor to OxyContin moving forward, Purdue explored other options to block its release and defend its market dominance through regulatory lobbying in addition to litigation.

### 3.4. 2003: Purdue attempts to block the release of generic oxycodone through litigation

Unable to resolve the disputes through satisfactory amendments, Endo appeared as a defendant in a patent suit brought by Purdue in the Southern District of New York. The case, which consolidated several related patent suits, was presided over by Judge Sidney H. Stein during a bench trial held for several weeks beginning in June 2003. Purdue’s suit rested on the claim that Endo had infringed on four patents related to OxyContin (Purdue Pharma LP v. Endo Pharms. Inc, 2004). Endo contended, in turn, that Purdue had engaged in inequitable conduct before the Patent and Trademark Office (PTO) by allegedly misrepresenting “the material fact that Purdue had ‘surprisingly discovered’ that its invention reduced the dosage range and eased titration in comparison to other opioid formulations. The misrepresentation lay in the failure to disclose material information inconsistent with these assertions”(Purdue Pharma LP v. Endo Pharms. Inc, 2004, pp. 18–19). Court documents reveal that Purdue’s ‘surprising discovery’ was more based on intuitive commonsense about the relative strength and pharmacokinetics of different opioid molecules and was not supported by any clinical data by the time they filed with the PTO (Purdue Pharma LP v. Endo Pharms. Inc, 2004, pp. 19–23).

In late 2003, after the bench trial had concluded but before the Court had issued its decision, Purdue sent out a call for proposals to several consulting agencies, including McKinsey, which it ultimately hired (Bogdanich & Forsythe, 2022). In the call, Purdue outlined its business position and what it aimed to gain through consultancy; although it had approximately $2 billion in yearly sales worldwide, 80% “come from one major product, OxyContin,” and that since OxyContin’s launch in 1995 the company “has not launched a major new product of its own research”(Purdue Pharma L.P., 2003). Considering research and development constraints, as well as “a number of market environment challenges resulting from increasing regulatory, competitive, and public health pressures (labeling, risk management, scheduling),” Purdue issued a proposal “[t]o establish a product finding, assessment, research and development system that will improve upon our historical success rate and make new products available to Purdue every year, either through in-house development or acquisition from third parties through licensing, acquisition or other partnering activities”(Purdue Pharma L. P., 2003).

Although the consultant hired would be charged with re-designing Purdue’s product pipeline and diversifying its portfolio, Purdue’s decision-making and governance structure, and its main source of revenue, OxyContin, were out of scope. Purdue wanted to mitigate its organizational and financial vulnerabilities in the wake of an unfavorable lawsuit outcome yet was reluctant to address its singular reliance on OxyContin for revenue, nor the leadership that allowed that reliance to take shape.

### 3.5. January 2004: Purdue’s initial effort to block generic oxycodone through litigation fails

In its decision issued January 5th, 2004, the Court ruled that while Purdue had proven “by a preponderance of evidence” that Endo had infringed on its patents, Endo had “proven by clear and convincing evidence that those patents are invalid due to Purdue’s inequitable conduct before the PTO,” thereby rendering those patents unenforceable (Purdue Pharma LP v. Endo Pharms. Inc, 2004, p. 23). The Court dismissed Purdue’s claims against Endo, declared several patents invalid, and further enjoined Purdue from enforcing those patents (Purdue Pharma LP v. Endo Pharms. Inc, 2004, p. 23).

### 3.6. 2004: Purdue pursues alternative strategies to protect OxyContin

On January 12th, 2004, Purdue filed a petition with the FDA to include a risk management program (RMP) in its approved labeling. Purdue presented the petition as an effort to educate providers and to reduce abuse and diversion of OxyContin through post-market surveillance. Pharmaceutical publications at the time, however, noted that including a risk management program as part of labelling would effectively postpone the approval of Endo’s generic, pointing to a similar effort by Roche regarding their acne drug Accutane (Unknown, 2004). In other words, other actors within the pharmaceutical industry viewed Purdue’s efforts as an attempt to ‘evergreen’ their product through other means, namely lobbying for changes in regulatory standards that would undermine their competitors, a phenomena well-documented by pharmaceutical industry scholars (Andrade et al., 2023; Eisenkraft Klein et al., 2025; Gagnon, 2013, 2021b; Hemphill & Sampat, 2012).

In January 2004, McKinsey staffers circulated a draft presentation that outlined their perception of Purdue’s situation, in response to its call for proposals to redesign the company’s product pipeline. The slide deck attached to emails indicated that Purdue was in a crisis and listed several domains that McKinsey was prepared to address (Zbar, 2004). By January 26th, 2004, McKinsey circulated a course correction plan for Purdue internally. This slideshow emphasized a dramatic “scaled reduction” of several projects and costs, including plant infrastructure and offices, drug safety and surveillance programs, medical education, and internal drug discovery and development, while maintaining development and marketing on OxyContin. The plan suggested these recommended actions would achieve “a higher ratio of direct project spend through consolidation/elimination”(Purdue Pharma L.P., 2004). By eliminating unnecessary costs, focusing on products already on, or close to, the market, and externalizing new drug development through licensing and partnerships, Purdue might be able to weather the loss of revenue it faced in the wake of a failed appeal that allowed Endo to market a generic version of OxyContin.

In a discussion document presented to Purdue, dated February 11th, 2004, McKinsey proposed that Purdue might become “Stronger than Before” by realigning its research and development priorities. McKinsey instructed R&D management to “aggressively pursue possible near-term structure and operations-related efficiencies” to reduce costs and rebuild a ‘war-chest’ that has largely been wasted in past years through unsuccessful internal drug development (Miller, 2004). This approach included selling off Purdue’s drug development portfolio, and if no buyer was found, ending its investments in new drug discovery entirely. However, McKinsey expressed cautious optimism about two new OxyContin reformulations (Miller, 2004). The first, OCX, was an oral “abuse-resistant” OxyContin formulation projected to launch in the 2nd quarter of 2009 with estimated peak sales of around $2.9 billion in the US. However, there was a potential risk that naltrexone leakage in some patients would reduce efficacy and create a regulatory hurdle (Miller, 2004). The second, an “abuse-resistant” formulation specific to the EU, had more modest sales estimates of $385 million, with risks pertaining to uncertain “Ability to demonstrate long-term efficacy and safety in both malignant and non-malignant pain patients”(Miller, 2004). In either case, reformulation of OxyContin as an abuse-deterrent was presented as a viable pathway for an otherwise unsustainable research and development program within Purdue.

### 3.7. 2005: Purdue investigates a partnership with Grünenthal to reformulate OxyContin

Given that McKinsey had proposed that in-house development should be scaled down, it became imperative for Purdue to work with external partners to develop reformulations. Internal emails show that, during its 2004 appeal against Endo, Purdue had begun negotiations with the German pharmaceutical company Grünenthal, particularly regarding the use of their abuse-deterrent technologies in a reformulated OxyContin (Ottimo, 2004a, 2004b). Work on developing a deal with the German company began in late 2004, with McKinsey supporting Purdue through financial evaluations and potential market scenario decision trees, often circulated in the form of extensive slide decks (Kraft, 2004; Ottimo, 2004a, 2004b; Shum, 2005b). At the end of 2004, Purdue made a formal proposal to Grünenthal that stressed the company’s critical interest in abuse-deterrent formulation technologies (Friedman, 2004). Under the proposed terms, Purdue would retain worldwide rights to an abuse-deterrent formulation version of OxyContin, and Grünenthal would enjoy a 10% royalty on worldwide sales as well as cash support for their research and development program (Friedman, 2004).

In early 2005, formal negotiations began on a co-development deal on an abuse-deterrent formulation of OxyContin (Shum, 2005a). The terms of this agreement, as reflected in draft documents circulated between companies, included key negotiations around intellectual property (Pâques, 2005). Although Grünenthal would have retained the intellectual property rights on their abuse-deterrent formulation technology and any improvements and inventions related to it, Purdue demanded “a first right of refusal for the abuse-deterrent formulation technology in other products since we could potentially develop the IP, fund the R&D, and build the brand equity”(Pâques, 2005). Grünenthal proposed a gradual phase-out of the non-abuse-deterrent formulation of OxyContin and its replacement with the abuse-deterrent formulation; however, the marketing staff at Purdue was hesitant (Pâques, 2005).

Purdue expressed strong interest in extending the patent life and protecting the value of OxyContin. In a January 11th, 2005, internal email sent during formal negotiations with Grünenthal, McKinsey consultant Brian Stolz indicated that Grünenthal had initially demanded a 50/50 profit split, working on the assumption that by incorporating their abuse-deterrent formulation technology Purdue would be able to “extend the exclusivity of Oxy[Contin] for about 10 years, and they think their drug can compete with Oxy[Contin] if we don’t launch with them”(Stolz, 2005). Stolz added that Purdue’s response to this proposal-cum-threat was negative, and that McKinsey (and by extension Purdue) viewed the value of abuse-deterrent formulations more broadly:

“G[rünenthal] thinks they can extend our patent. I think we are at odds with this assessment since even if the abuse-deterrent formulation technology extends the patent life of our new product under the abuse-deterrent formulation patents, Oxy in its current form will go generic. Unless the FDA requires all Oxycodone formulations to have abuse-resistant characteristics, we don’t think they will keep generics from entering the market. The idea that abuse-deterrent formulation brings half of the value to the transaction seems very far-fetched.“

(Stolz, 2005)

Purdue concluded that although an abuse-deterrent formulation reformulation of OxyContin would extend its patent life, that reformulation would do little in the long term to protect Purdue’s sales unless some kind of regulatory action curtailed the generic market for non-abuse-deterrent formulation varieties (Stolz, 2005). In other words, absent governmental intervention to change the competitive landscape, the return on investment from reformulation was limited.

### 3.8. 2006: Purdue wins its appeal against Endo International

On February 1st, 2006, the US Appellate Court ruled in Purdue’s favor, declaring that Purdue’s patents were enforceable, and that Endo had been infringing on those patents with its generic (Purdue Pharma LP v. Endo Pharmaceuticals Inc., 2006). The Court vacated the SDNY trial decision, returning the case for reconsideration. Purdue and Endo then entered settlement negotiations. On August 28th, 2006, Purdue CEO Michael Friedman issued a statement declaring victory over Endo as part of an official press release (Bannon, 2006). As part of the final agreement reached by the two companies, Endo admitted infringement on Purdue’s rightfully held patents and ceased all sales and promotion of its generic by December 31st, 2006. CEO Friedman recalled the strategy outlined by Sackler nearly a decade prior, stating:

“We will continue to defend our invention of that medicine against all infringers. Our dedication to serving both healthcare professionals and patients with innovative prescription and non-prescription products has never waned during the long course of this litigation, and we look forward to maintaining that commitment long into the future.“

(Bannon, 2006)

In the wake of the decision, negotiations with Grünenthal faltered. As later court documents show, Purdue backed out of negotiations for co-development and instead opting to license Grünenthal’s intellectual properties to develop their own abuse-deterrent mechanisms, paying Grünenthal millions in royalties over several years (Purdue Pharma L.P. et al. v. Teva Pharmaceuticals, 2014).

### 3.9. 2007–2013: The US regulatory system changes, and Purdue resurrects the idea of an abuse-deterrent formulation

In 2007, Congress passed the Food and Drug Administration Amendments Act (FDAAA), which allowed the agency to conduct surveillance and evaluation of approved drugs after they entered the market, as well as existing pre-market clinical trial data (Avorn et al., 2018). In 2012, the FDA codified this authority through a class-wide Risk Evaluation and Mitigation Strategy (REMS) program for long-acting prescription opioids, effectively raising the hurdle manufacturers had to clear to prove the safety and efficacy of their drugs (Cepeda et al., 2017; Nelson & Perrone, 2012; Stanos, 2012). In the late 2000s, researchers suggested abuse-deterrent formulations were a possible means for addressing the opioid crisis based on the Talwin experience of the 1980s (Cicero & Ellis, 2015; Katz et al., 2007; Mastropietro & Omidian, 2015a, 2015b, Schaeffer, 2012). Though some scientists promoting ADFs as a solution have well-documented industry ties (i.e., Katz et al., 2007; Webster, 2009), this connection was not universally true and it seems that at least some independent researchers saw genuine promise in ADF opioids as a public health strategy.

Purdue’s interest in abuse-deterrent formulations reappeared when the regulatory climate shifted. Increased federal scrutiny required opioid manufacturers to prove safety and provide strategies to prevent abuse or diversion prior to product marketing, with Purdue officials citing the FDA’s “increasingly risk conservative position” as a cause for concern (Landau, 2008). Redeveloping existing opioids using abuse-deterrent technologies and delivery systems was one strategy to address these benchmarks (Lenz, 2009a; Tramontin, 2009). In consultation with the FDA, Purdue voluntarily reformulated OxyContin to be “abuse resistant” in August of 2009, replacing the original formulation by early 2010. The reformulation that ultimately reached market was one that McKinsey had identified as worth retaining in early 2004: OCX, now renamed OTR (Oxycontin Tamper-resistant) (Buchhalter, 2009; Carney, 2009; Lenz, 2009b; Rappaport, 2009).

In August 2010, Purdue Pharmaceuticals quietly introduced a reformulation of OxyContin (oxycodone) to the US market (Schaeffer, 2012). Earlier that year, the FDA had approved an abuse-deterrent formulation of OxyContin that was purportedly intended to reduce the diversion of the potent long-acting opioid, and reduce misuse, overdoses, and deaths (Beachler et al., 2022). Atypically, the introduction did not involve the customary price changes, press releases, or notifications to patients and prescribers (Beachler et al., 2022; Cicero & Ellis, 2015). Shortly after approval, Purdue, in consultation with the FDA, ceased shipments of the original extended-release formulation of OxyContin with the intent of replacing it with the new abuse-deterrent formulation (Alexander et al., 2014; Schaeffer, 2012). By December 2010, an estimated 90% of OxyContin prescriptions dispensed were for the abuse-deterrent formulation; by December 2011, 99% of dispensed OxyContin prescriptions were for the abuse-deterrent formulation (Beachler et al., 2022, p. 396).

In 2015, the FDA issued final guidance on the evaluation and labeling of abuse-deterrent formulation opioids, which the agency defined succinctly as drugs with “properties shown to meaningfully ***deter*** abuse, even if they do not fully ***prevent*** abuse” (FDA Center for Drug Evaluation and Research, 2015, p. 2). The FDA identified abuse-deterrent formulations as a critical means to “address the problem of opioid abuse while [ensuring] that patients in pain have appropriate access to opioid products”(FDA Center for Drug Evaluation and Research, 2015, p. 3). Purdue’s development an abuse-deterrent formulation for OxyContin, and its release of this reformulation had preceded these regulatory mandates and guidance, as well as the release of other abuse-deterrent formulation opioids. The FDA’s previous experience with Talwin suggests regulators were at least aware of abuse-deterrence as a concept prior to Purdue’s reformulation of OxyContin. It is entirely possible that Purdue’s initially became aware of this reformulation through conversation with the FDA amidst the opioid crisis. It is also possible Purdue retained some institutional memory regarding Talwin’s reformulation, given their activity in the pain management field during the 1980s. Regardless of where the idea to reformulate OxyContin originated, Purdue stalled development and release until the intersection of patent considerations and the regulatory environment made abuse-deterrent reformulation the most advantageous.

## 4. Discussion

Our qualitative archival analysis of industry documents revealed that Purdue’s initial interest in an abuse-deterrent formulation of OxyContin was not motivated by regulatory pressure or concern for public health. Instead, Purdue considered developing an abuse-deterrent formulation as a strategy to support its patent litigation and to forestall the release of a competing generic drug. After a federal court ruled that Purdue’s patents were unenforceable in 2004, the company began working with McKinsey to extend or renew OxyContin’s patent protection by incorporating abuse-deterrent technologies. When Purdue won an appeal on its patent litigation in 2006, it deprioritized its development of an abuse-deterrent formulation. These earlier plans, however, left the company well-positioned to respond to new regulations in the late 2000s. Purdue delayed the actual release of its abuse-deterrent formulation until the regulatory environment changed in a way that would mean releasing the reformulation could prevent the release of generics (Lenz, 2009a, 2009b). Although Purdue suggested later that abuse-deterrent formulations were critical to mitigating opioid abuse, internally, Purdue and McKinsey viewed them primarily as a strategy to protect corporate profits (McKinsey, 2004; Stolz, 2005). This corporate understanding is consistent with clinical research that suggests the public health value of abuse-deterrent formulations is limited, given that there is little evidence that opioids are effective for treating chronic pain or that opioids are only harmful when people tamper with them (Becker & Fiellin, 2017). Perhaps tellingly, while Purdue withdrew its original formulation OxyContin and replaced it with an abuse-deterrent formulation in the United States in 2010, it waited until 2012 to introduce the ADF in Cananda, when its patents on OxyContin expired in that country. With its market share declining, Purdue lobbied during parliamentary debates to advocate mandating abuse-deterrent formulation opioids, which offered to restore Purdue’s advantageous exclusivity (Eisenkraft Klein et al., 2025).

Our case study on Purdue’s early interests and development of abuse-deterrent formulation opioids advance the literature on commercial determinants of health by introducing an analysis of competitive markets dynamics and strategies corporations use to gain an edge and disadvantage their competitors, including through aggressive patent litigation and strategic regulatory lobbying. Our study, however, is not the first to observe these dynamics. Numerous scholars have pointed out how intellectual property regimes shape corporate strategy, competitive behavior, and relations with consumers. For example, much has been written about the practice of evergreening in the pharmaceutical industry, which Feldman defines as “artificially extending the life of a patent or other exclusivity by obtaining additional protections to extend the monopoly period”(Feldman, 2018, p. 596). Evergreening strategies include those that appear in our results. For example, evergreening can be achieved by filing for new patents on old drugs that is, finding new things about the existing drug to patent without necessarily changing it, or modifying formulation or by patenting specific features of production and manufacturing processes. Evergreening can also be achieved by minimally reformulating existing drugs, developing new dosage schedules, or creating new combination drugs that can secure patent protections without affecting therapeutic value, while withdrawing old formulations (whose patent protections face expiry) from the market (Feldman, 2018, pp. 601–602). These strategies allow patent-holding companies to leverage intellectual property law to block generic competitors, disadvantage new organizational entrants to the market, who often do not hold patents themselves or have the funds to license intellectual property, and overall depress therapeutic innovations by favoring minimalistic reformulation of existing drugs, which conceivably cost less to research and develop, over developing entirely new drugs, therefore providing a greater return on investment for pharmaceutical companies (Andrade et al., 2023; Feldman, 2018; Gagnon, 2013).

Political economists of the pharmaceutical industry have critiqued the pernicious effect the current intellectual property regime has had both on market competition and patients. In the words of one pharmaceutical executive, the current model is one where new drugs are “slightly-different-marketed-like-hell,” where minimal changes to existing drugs, often with few to no therapeutic improvements, are incentivized over genuine innovation and therapeutic breakthroughs (Gagnon, 2013, p. 571). The value of major pharmaceutical companies, such as Pfizer, thereby become predicated not on genuine productivity or innovation, but rather their “capacity to restrain others’ production through patent monopolies” and leverage their patent portfolio’s to create “patent gridlocks” and “patent thickets,” that stifle new drug development and competition through the threat of litigation, a situation Heller and Eisenberg term the “Tragedy of the Anticommons” (Gagnon, 2021; Heller, 2010; Heller & Eisenberg, 1998).

Consequently, research and development programs progress with eventual marketing strategies and sales in mind, operating in tandem with corporate ‘ghost-management’ of scientific knowledge production about new drugs (Gagnon, 2013, 2021b; Sismondo, 2007; Sismondo & Bernisson, 2024). Existing intellectual property laws systemically incentivize minimal innovation and jealous defense of existing patents over and at times at odds with patient safety. Gagnon terms the entire structural arrangement that incentivizes and allows for these practices as “institutional corruption”(Gagnon, 2013), while specifically framing corporate activities to secure and maintain an advantageous position within this structural arrangement, through scientific production and regulatory manipulation, as ‘technical capture’(Gagnon, 2021a, 2021b). As Gagnon rightly points out, pharmaceutical corporations are not necessarily to blame for this corruption, nor can they be blamed for their efforts to capture scientific and regulatory institutions (Gagnon, 2013). They are simply working within the possibilities of existing law and regulation to best secure profit in a putatively competitive environment; it just happens that the consequence of multiple corporations following this strategy is that innovation stagnates, clinical knowledge skews, and patients suffer.

Insights from political economists of the pharmaceutical industry help to contextualize our findings specifically, and more generally help advance commercial determinants of health research by focusing on how law, regulation, and competition might shape the corporate activities that harm public health. For example, understanding how intellectual property and patent disputes shaped the development of new products in the tobacco industry (from filters, to additives, to e-cigarettes) may help to better situate and explain the historical trajectory of tobacco use trends and attendant harms (Cataldo & Malone, 2008; Fooks et al., 2011; Peeters & Gilmore, 2013; Proctor, 2012). Relatedly, the patentability of novel molecules, synthesis techniques, and production processes, within the context of an industry wide race for dominance, may shed light on how harmful chemicals found their way into ubiquitous products, from pesticides to soft drinks (Conis, 2022; Fazzino et al., 2024; Kearns et al., 2018; Nguyen et al., 2019; Vainio, 2020). Industry documents allow for a strategic look into how these competitive dynamics motivate and shape corporate strategy, communications, knowledge production and management, promotion, and ultimately harm to consumers. Commercial determinants of health scholars using industry documents could thus benefit from insights offered by political economists and other critical researchers of the pharmaceutical industry.

## 5. Limitations

Our study has limitations. While our findings describe motivations underlying the development of Purdue’s abuse-deterrent formulation, retrospective review cannot establish direct causality or disprove alternative potential explanations. The data collection and analysis also reflect an incomplete record; although OIDA included over 5 million documents at the time of data collection, it does not contain all documents produced by opioid manufacturers, and unreleased documents could be relevant to understanding the history and development of abuse-deterrent formulations. Supplementary documents and secondary literature also constitute an incomplete record, as they represent only the documents that were cited in court decisions, finalized regulatory documents, and published research. Finally, the motivations and actions of Purdue may not necessarily be reflective of other companies, nor does the case of its ADF development in the United States necessarily mirror ADF development in other countries. Future research could expand on this work by considering the abuse-deterrent formulations developed by other companies, reviewing additional documents as OIDA continues to expand, or employing new research strategies to interpret a larger set of documents.

## 6. Conclusions

Assumptions that pharmaceutical companies have acted out of public health beneficence or regulatory compliance may obfuscate underlying profit motives that drive product planning, development, and marketing. Industry documents research allows for analysis of pharmaceutical companies’ internal motivations for product development, which often contrast with motivations and promise of benefits presented to regulators and the public. Our findings suggest the development of abuse-deterrent formulations may be a form of pharmaceutical “evergreening,” a process whereby minor, iterative formulation changes allow for the indefinite extension of patents on drugs that would otherwise, by statute, become generic (Abbas, 2019; Andrade et al., 2023; Feldman, 2018; Hemphill & Sampat, 2012), while offering limited protection against health risks and distracting healthcare providers and policymakers from identifying more effective regulations (Becker & Fiellin, 2017). Ultimately, a regulatory focus on product reformulation as a strategy to prevent misuse, abuse, and overdose may undermine public health by favoring solutions preferred by opioid manufacturers rather than what is in the best interest of patients and people experiencing substance use disorders.

## Data Availability

The data supporting the conclusions of this article are publicly available at the University of California Industry Documents Library. https://www.industrydocuments.ucsf.edu/.
